# Nanodiamond embedded polyaniline/polyvinylidene fluoride nanocomposites as microfiltration membranes for removal of industrial pollution

**DOI:** 10.1039/d3ra05351b

**Published:** 2023-10-05

**Authors:** Asima Siddiqa, Abdul Majid, Farhat Saira, Saima Farooq, Rumana Qureshi, Sara Qaisar

**Affiliations:** a Nanoscience and Technology Division, National Centre for Physics Islamabad Pakistan a.sam.malik@gmail.com fsghaus@gmial.com; b Department of Chemistry, Quaid-i-Azam University Islamabad Pakistan; c Department of Biological Sciences &Chemistry, College of Arts and Science, University of Nizwa Nizwa-616 Oman

## Abstract

Membrane fouling remains a challenge to the membrane technology. Herein, we report the fabrication of composite membranes of polyaniline/polyvinylidene fluoride (PANI/PVDF) blended with nanodiamond (ND) with improved antifouling properties. The designed membranes were characterized by XRD, FTIR and SEM techniques. Characterization analysis revealed that addition of ND has maintained the structural integrity and porosity of composite membranes. The membrane permeation and antifouling performances were tested for hydrophilicity, porosity, pure water flux, shrinkage ratio, salt rejection of zinc acetate and copper acetate, and their fouling recovery ratio (FRR) measurements. A high solvent content ratio of 0.55 and a low shrinkage ratio of <12% due to enhanced hydrophilicity and porosity of the composite membrane with fouling-recovery of membranes to 88% were achieved. Separation of copper and zinc ions from aqueous solution was achieved. These findings imply that ND-based PANI/PVDF composite membranes can effectively serve as microfiltration membranes in industrial and municipal wastewater treatment.

## Abstract 2

Nanotechnology has a solution for membrane fouling which remained a challenge to the membrane technology.

## Graphical abstract

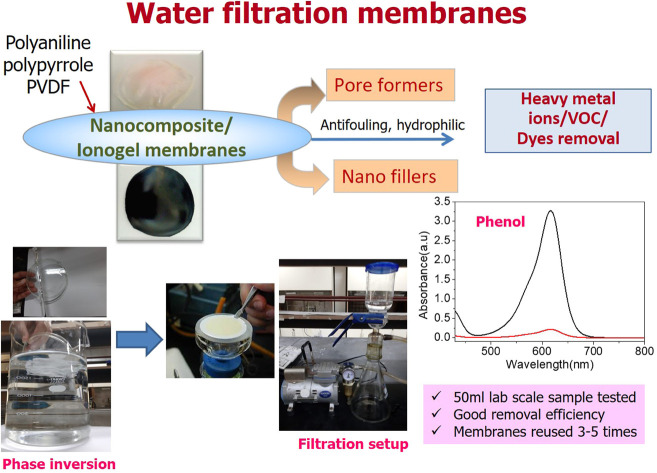

## 1. Introduction

Rapidly increasing rates of the world's population and industrialization together with prevalent climatic changes, have aggravated water scarcity and contamination across the globe.^1,2^ Alarmingly, around one-fifth of the world's population is facing a severe water shortage in terms of limited access to clean and/safe drinking water resulting in a significant number of deaths every year.^1,3^ Thus, critical global water demand has challenged the scientific community to develop cost-effective and efficient technologies for water production and/or to recycle high-quality water.

Of all existing water treatment technologies such as adsorption, ion exchange mechanism, and precipitation reactions,^1,2^ the membrane-based filtration technologies *i.e.*, microfiltration (MF), ultrafiltration (UF), nanofiltration (NF), and reverse osmosis (RO), have been acknowledged as the most cost-effective, environmentally friendly, and technologically-matured.^4–6^ Their widespread applications include, but are not limited to, desalination, brackish water softening, wastewater treatment, industrial water discharge decontamination, *etc.*^7–9^ At present, polymeric membranes have received tremendous attention due to their unique characteristics including, interconnected pore structure, high surface area, flexibility, and relatively low-cost processing.^8,10^ Despite the outstanding features of membrane-based technologies, they suffer a critical limitation, membrane-fouling, due to their hydrophobicity and/or interfacial structure, which profoundly compromises the performance and lifespan of the membranes.^3,11–13^ Over the past decades, enormous efforts have been made to alleviate the membrane-fouling issue including surface modification by grafting and coating, bulk modification by mixing and blending hydrophilic polymers,.^14–18^ The rationale behind membrane modification is to enhance the water permeance across the membrane maintaining a high solute-rejection rate without compromising the active membrane area.

Polyvinylidene fluoride (PVDF) has been acknowledged as an excellent material in membrane sciences due to its incredible properties, such as remarkably high thermal and mechanical stability, chemical resistance, and exceptional membrane forming abilities.^19,20^ At the same time high hydrophobicity of PVDF compared to other polymer membranes makes it vulnerable to fouling which needs to be addressed. Numerous efforts have been devoted for antifouling of PVDF membranes *via* blending with hydrophilic nanofiller/polymers, chemical oxidation, plasma treatment, *etc.* For instance, Choi *et al.*^21^ reported the modification of PVDF membrane *via* blending with poly(ethylene glycol) methyl ether methacrylate (POEM) graft co-polymer microfiltration membrane and investigated their antifouling properties. The grafted membranes exhibited no irreversible fouling during filtration of different foulants *i.e.* bovine serum albumin, sodium alginate, and *E. coli* broth due to surface hydrophilicity of POEM polymer compared to the pristine PVDF membrane. Yoon *et al.*^22^ investigated the surface modification of polyethersulfone electrospun filtration membrane by oxidation process using ammonium persulfate and noticed enhanced hydrophilicity and filtration flux of the modified membrane. Nasreen *et al.*^23^ prepared electrospun nanofibrous membranes *via in situ* polymerization of PVDF with hydroxyethylmethacrylate (HEMA) followed by coating with surface-charge chitosan polymer. The observed comparatively better flux and recovery ratio of PHEMA electrospun membranes due to HEMA's hydrophilic nature compared to PVDF membranes. The PVDF-based membranes mixed cellulose esters (MCE) and polyethersulfone (PES) have been used for activated sludge treatment by Fang *et al.*^24^ Their findings showed that pore-fouling was affected by the hydrophilicity, microstructure, and pore openings of the composite membrane. These results, among various other studies,^25,26^ signify the concept of PVDF membrane modification *via* polymer blending, composite antifouling membranes, as a peculiar domain of membrane technology research. The incorporation of hydrophilic polymer into PVDF matrix noticeably enhances the composite membrane durability and flux rate by tuning the porosity of the membrane.^19,20,27^

Among various hydrophilic polymers, polyaniline (PANI) is reported to have profoundly high hydrophilicity, permeability, stability and porosity.^28–31^ PANI-blended membranes have been reported to exhibit higher permeability and antifouling properties due to their hydrophobicity.^32,33^ Another strategy to enhance antifouling membrane properties is the incorporation of nanomaterials, nanofillers, in the polymer composite membranes which significantly affect the physiochemical characteristics of matrix material.^2,15,17^ Nanofillers adhere to the polymer matrix *via* chemical bonding, increasing the polymer-filler phase compatibility which results in dramatic change of polymer blend behavior.^34^ The nanofillers including alumina, palladium, silica, gold, graphene, titania, carbon nanotubes, graphene oxide and nanodiamonds *etc.* have been extensively used to fabricate polymer nanocomposite membranes for water filtration.^35–37^ Recently, nanodiamonds (NDs), one of the most attractive allotropes of carbon, has emerged as a functional nanofiller with outstanding properties such as high mechanical and thermal stability, homogenous size distribution, non-toxicity, and high surface area with tunable surface structures.^38,39^ ND blended membranes can dramatically reduce the irreversible fouling ratio about 3–4 times compared to the pristine membrane and enhance their structural stability and surface functionalities, making them highly desirable in a wide range of applications.^40–42^ Previously we had reported the hydrophilicity of nanodiamonds in PDVF/ND composite microfiltration membranes.^43^ It was observed that a higher content level of ND (5%) has surprisingly enhanced the water flux, water content and porosity of the composite membrane.

Undoubtedly, polymer blending and nanofillers' incorporation into the PVDF matrix is the novel strategy to achieve the desirable antifouling properties of hydrophobic PVDF membrane. Here we report the fabrication of poly(vinylidene fluoride)-polyaniline (PVDF–PANI) nanocomposite microfiltration membranes impregnated with nanodiamond (ND) fillers (1–5 wt%) *via* solution casting method. We have presented the physical and chemical properties of PVDF-PANI polyblend and ND-incorporated PVDF-PANI nanocomposites microfiltration membranes which propose these high-performance membranes may open up new avenues for engineering of nanofabricated membrane materials for wide range of applications.

## 2. Experimental section

### 2.1 Materials

Polyvinylidene fluoride (PVDF) powder (*M*_w_ ∼530 000, density 1.74 g cm^−3^) was purchased from Sigma Aldrich USA. Polyaniline (PANI) powder (*M*_w_ ∼15 000, density 1.329 g cm^−3^) was obtained from BDH Laboratories. Commercial nanodiamonds (NDs) (% purity 95, 50 nm average size) were taken from Heyuan Zhong Lian Ltd, China. These NDs were functionalized prior to their utilization for fabrication of membrane. The entire reagent package including sulfuric acid (H_2_SO_4_ ≥97.5%), hydrochloric acid (HCl ≥37%), nitric acid (HNO_3_ ≥70%), *N*,*N*-dimethyl formamide (DMF ≥99%), sodium hydroxide (NaOH ≥99%), ethanol (C_2_H_5_OH ≥99%), acetone (C_2_H_6_O ≥99%), zinc acetate and copper nitrate were obtained from Sigma Aldrich USA with a purity of 99%.

### 2.2 Functionalization of nanodiamonds

Surface functionalization of nanodiamonds was performed according to the following procedure as reported earlier.^40^ In the first step NDs were oxidized with the mixture of H_2_SO_4_ and HNO_3_ (3 : 1) at 90 °C for 1 hour under constant stirring. Subsequently the solution was filtered and washed repeatedly with deionized water until pH maintained to 7. In the second step, treated NDs were poured into a mixture of H_2_SO_4_ and HNO_3_ (9 : 1) and stirred for 3 days at 90 °C. The NDs obtained were again filtered and washed with deionized water until the pH reached to neutral. At the end, resulting material was treated with 0.1 M NaOH and 0.1 M HCl, respectively, followed by washing with deionized water. The obtained slurry was dried in vacuum oven for 24 hours at 100 °C to achieve surface functionalized NDs.

### 2.3 Fabrication of composite membranes

PVDF-PANI and PVDF-PANI/NDs composite blend membranes were fabricated prepared *via* solution casting method.^44^ Desired amounts of PANI and PVDF in ratio 2 : 1 (wt%) were added in the DMF solvent and sonicated for about half an hour at 60 °C for well dispersion. After complete dispersion, the solution was left still for 1 hour to remove the trapped air bubbles. The solution was then casted in the vacuum oven at 80 °C until a film is obtained.

For the synthesis of PVDF-PANI/NDs nanocomposite membranes, PANI and PVDF ratio was kept constant 2 : 1 while nanodiamonds were varied from 1–5 wt% and were labeled as 1ND-PANI, 2ND-PANI, 3ND-PANI, 4ND-PANI, and 5ND-PANI.

### 2.4 Membrane characterization

To ascertain the structural features of the composite membranes X-ray diffraction (XRD) analysis was conducted by Panalytical 3040/60 X′ Pert PRO diffractometer in the range of 10° to 80°. FTIR analysis was performed over the scan range of 500–4000 cm^−1^ using 1000 PerkinElmer. The surface morphology was observed by Scanning electron microscopy (Quanta 600F). To evaluate the thermal stability and phase transformation of the composite membrane samples, thermal gravimetric analysis was employed using TGA/DA PerkinElmer USA in the temperature range 50–800 °C with scan rate was 10 °C min^−1^.

### 2.5 Membrane permeation performances

#### 2.5.1 Water flux study

A vacuum filtration setup was employed for estimation of pure water flux of the membranes, as the amount of water passing across the membrane per unit time per unit area under transmembrane pressure. The membrane was subjected to the pure water flux estimation at trans membrane pressure of 0.2 bar. Pure water flux was calculated under steady-state flow using following equation;^45^1
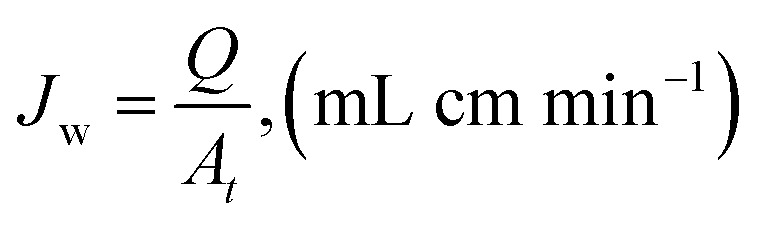
where *J*_w_ is pure water flux (mL cm min^−1^), *A* is membrane area (cm), *t* is filtration time (min) and *Q* is amount of permeate during filtration time (mL).

#### 2.5.2 Membrane porosity

The porosity of composite membranes was determined by measuring dry and wet membrane weight of pieces of membrane (1 cm dimension). In the next step, membrane was soaked in distilled water for 24 h and weighed out by mopping with blotting paper. Then wet membrane was dried at 70 °C in oven overnight and weighed again. From two membrane weights (wet and dry), the porosity of membrane were determined using formulae:^46^2
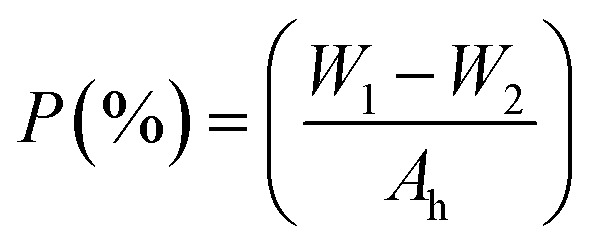
where *P* is porosity (g cm^−2^) of membrane, *W*_1_ is wet membrane weight (g), *W*_2_ is dry membrane weight (g) and *A*_h_ is area of wet membrane (cm^2^).

#### 2.5.3 Solvent uptake measurements

For the solvent content/uptake estimation, the membrane was cut into four square pieces having dimension and an area of 1 cm and 1 cm^2^, respectively. These four different pieces of membrane were separately soaked in each solvent (water, methanol, ethanol, and propanol) for 24 h and weighed by mopping with blotting paper. The wet membrane was dried in oven at 70 °C overnight and the weight of dried membrane sample was measured. The equation for the calculation of water uptake is given as follow:3
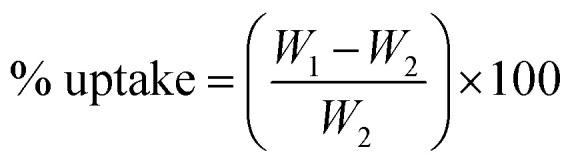
where, *W*_1_ and *W*_2_ are the weights (g) of the wet and dry membrane, respectively.

#### 2.5.4 Membrane shrinkage ratio (%)

For the shrinkage ratio estimation, a piece of wet membrane was taken and its length and breadth are determined. The piece was then dried at 100 °C overnight followed by re-measurements. From these values the shrinkage ratio was then calculated as:4
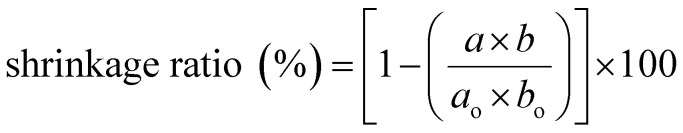
where *a* and *b* are the length and width of dry membrane and “*a*_o_” and “*b*_o_” are the length and width of wet membranes respectively.

### 2.6 Membrane antifouling performance (salt rejection and fouling recovery ratio)

The salt-rejection factor determines the amount of the salt retained by a membrane or in other words, it accounts for the capacity of a membrane to reject the undesired salt/compound from the feed mixture. On other hand, membrane fouling refers to the blocking of the pores in membrane due to the collection of solutes from the feed solution during filtration process. Hereafter, the fouling ratio accounts for antifouling ability of a membrane to fouling or % reduction in fouling. The salt rejection ratio of membranes was tested against heavy metals such as copper acetate and zinc acetate solutions (0.1 M each), model salts, under trans-membrane pressure of 0.2 bar. The concentration of salt was determined in the feed and filtrate was determined using conductivity meter as per following formula;

5 Salt rejection (SR) = 1 − *C*_p_/*C*_f_

where *C*_p_ and *C*_f_ are the salt concentration in filtrate (permeate) and salt concentration of feed, respectively.

Membrane antifouling performance was tested by previously reported method.^47^ After measuring pure water flux (*J*_w_1__) of membrane at trans-membrane pressure of 0.2 bar, 0.1 M aqueous solution of each model salt (copper acetate and zinc acetate) was individually filtered through membranes for 30 min at same pressure. Afterward the membranes were flushed with water under identical conditions of time and pressure and pure water flux (*J*_w_2__) was measured. The antifouling recovery ratio (FRR) of membranes was then measured using following relation:

6 Fouling recovery ratio (FRR) = *J*_w_2__/*J*_w_1__ × 100

Here *J*_w_2__ is the flux of cleaned membrane and *J*_w_1__ is the flux of pure membrane.

## 3. Results and discussion

### 3.1 XRD of composite blend membranes

**Fig. 1:**
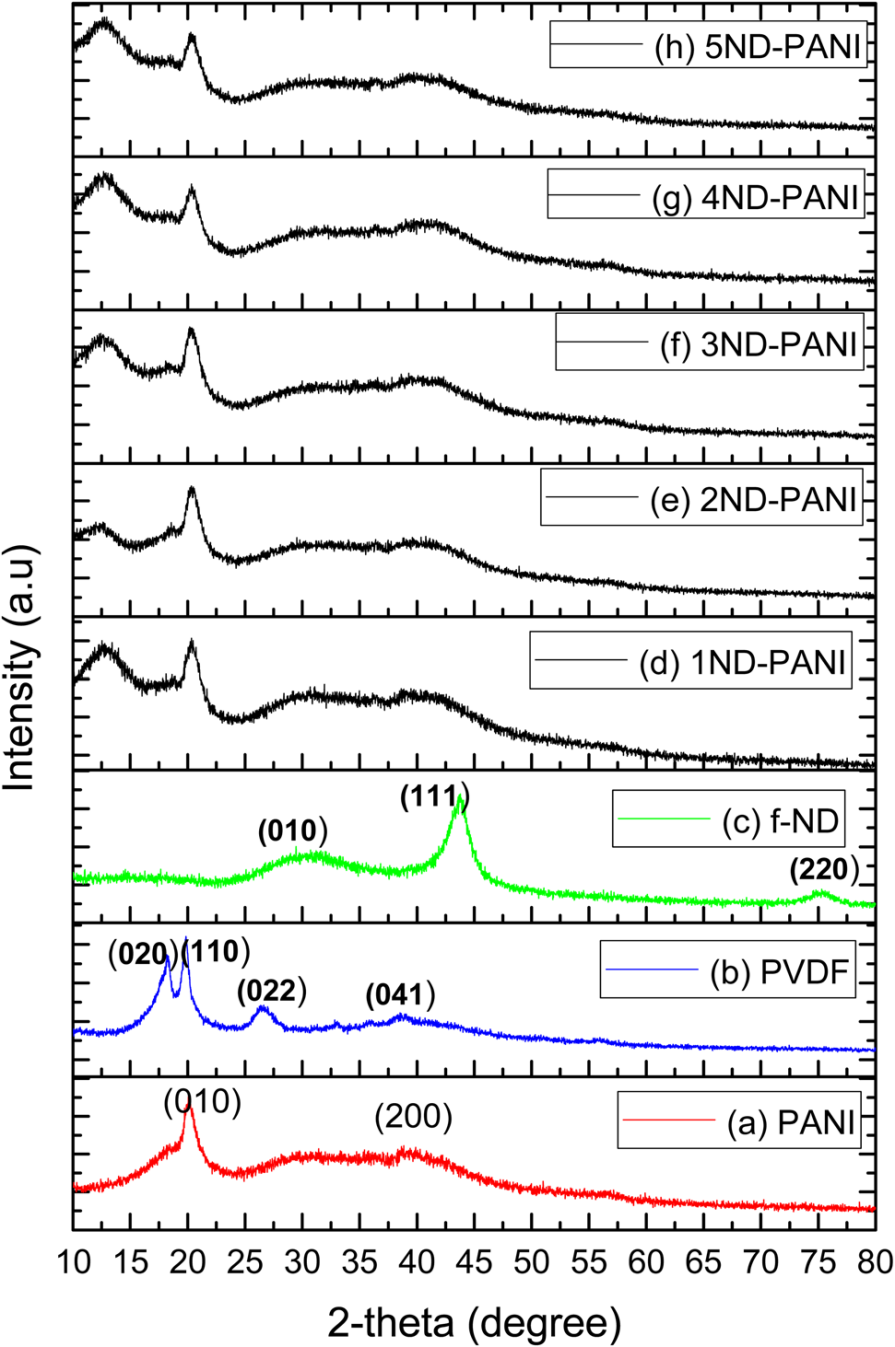
XRD patterns of composite blend membranes (a) pristine PANI (b) pristine PVDF (c) functionalized ND (f-ND) (d) 1ND-PANI (e) 2ND-PANI (f) 3ND-PANI (g) 4ND-PANI (h) 5ND-PANI.

XRD patterns of the synthesized materials are shown in Fig. 1. As shown in Fig. 1a, PANI with diffraction peaks at 20.05 and 40.02° was accorded to the pseudo-orthorhombic phase.^48^ The XRD pattern of pristine PVDF (Fig. 1b) with diffraction peaks 18.4, 20.18, 26.5, 33.91, 38.01, and 40.41° was corresponded to β-phase-PVDF with monoclinic structure.^49^Fig. 1c demonstrated the diffraction peaks of functionalized ND at 32.1, 43.2 and 75.0° corresponding to sp^3^ hybridized carbon structure.^43^ Similar observations have been reported in the literature.^43,48,49^ The XRD profile of PANI/PVDF composite membranes doped with varying concentration of NDs from 1–5% (Fig. 1d–h) illustrates broad diffraction peaks between 20°-40° due to perpendicular and parallel periodicities of the PANI polymer chain. Upon incorporation of NDs in these PANI/PVDF composite membranes, the characteristic peaks of NDs are observed at 20.5 and 43.01° while a broad peak is also observed at 38.72° indicative of β-phase of PVDF. These observations indicate that addition of ND has maintained the structural integrity of the polymer blend. Moreover, with the increase in the concentration of NDs increase in area under the diffraction peaks and intensity are observed. This could be attributed to the reason that NDs have acted as filling agent in these composites resulting in high mechanical strength and formation of defects.^40^

### 3.2 FTIR of composites membranes

**Fig. 2:**
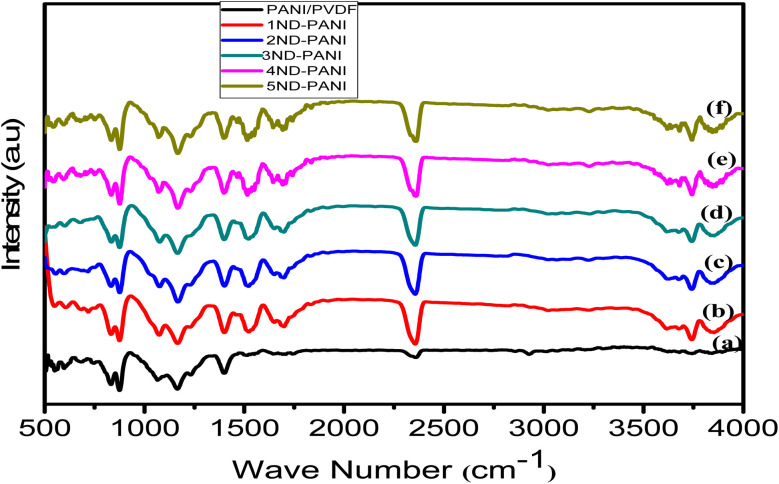
FTIR of microfiltration membranes (a) undoped PANI/PVDF (b)1ND-PANI (c)2ND-PANI (d) 3ND-PANI (e) 4ND-PANI (f)5ND-PANI.

Fig. 2a illustrates the FTIR spectrum of undoped PANI/PVDF polyblend. The absorption band appearing in the region of 825–870 cm^−1^ corresponds to C–F stretching vibration of PVDF, whereas the bands around 1082 and 1155 cm^−1^ represent in-plane bending vibrations of aromatic C–H group and C–C band vibrations, respectively. The characteristic band of C–N^+^ stretching vibrations of PANI appears at around 1409 cm^−1^ and C–H vibration of CH_2_ group is represented by appearance of absorption band at around 2366 cm^−1^.^50^Fig. 2b–g presents FTIR spectra of PANI/PVDF polyblend with varying amounts of ND (1–5 wt%) which resemble the absorption pattern of the polymer backbone. Besides, appearance of new absorption bands around 1600 cm^−1^ and ∼3700 cm^−1^ corresponding to C=O of COOH group and OH group, respectively confirm the inclusion of ND in PANI/PVDF polyblend. These findings are consistent with previously reported literature.^42,43,51^

### 3.3 Scanning electron microscopic (SEM) of composite microfiltration membranes

**Fig. 3:**
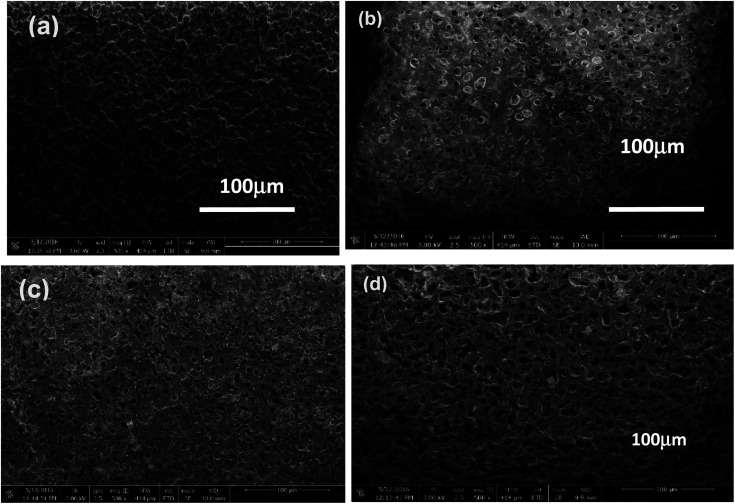
SEM images of (a) undoped PANI/PVDF polyblend (b) 1ND-PANI/PVDF (c) 3ND-PANI/PVDF (d) 5ND-PANI/PVDF.

Scanning electron microscopic (SEM) micrographs of undoped PVDF/PANI membrane, 1 wt% nanodiamonds (1ND-PANI), 3 wt% nanodiamonds (3ND-PANI) and (5ND-PANI) are shown in Fig. 3. The SEM image of composite membrane with 5 wt% of ND is porous as compared to other membranes in series. The porosity as well as pore size has increased upon the successive inclusion of nanodiamonds depicting the role of nanofiller as a pore forming agent. Moreover, the surface is found to be homogenous and no segregates of nanodiamonds are observed which proves an efficient interaction of the matrix (PANI/PVDF) with the filler (NDs) material.^52^ This establishes the fact that NDs are of smaller size and are well embedded in the polyblend matrix whereas; an increase in the pore size is attributed to repulsions between the polymeric chains and/or matrix and filler materials. It has been reported that electrostatic repulsions between polymer chains hinder their coagulation resulting in the formation of wide pores. The addition of ND might have caused the pattering of the pores.^43^

### 3.4 Thermal gravimetric analysis (TGA) of composite microfiltration membranes

**Fig. 4:**
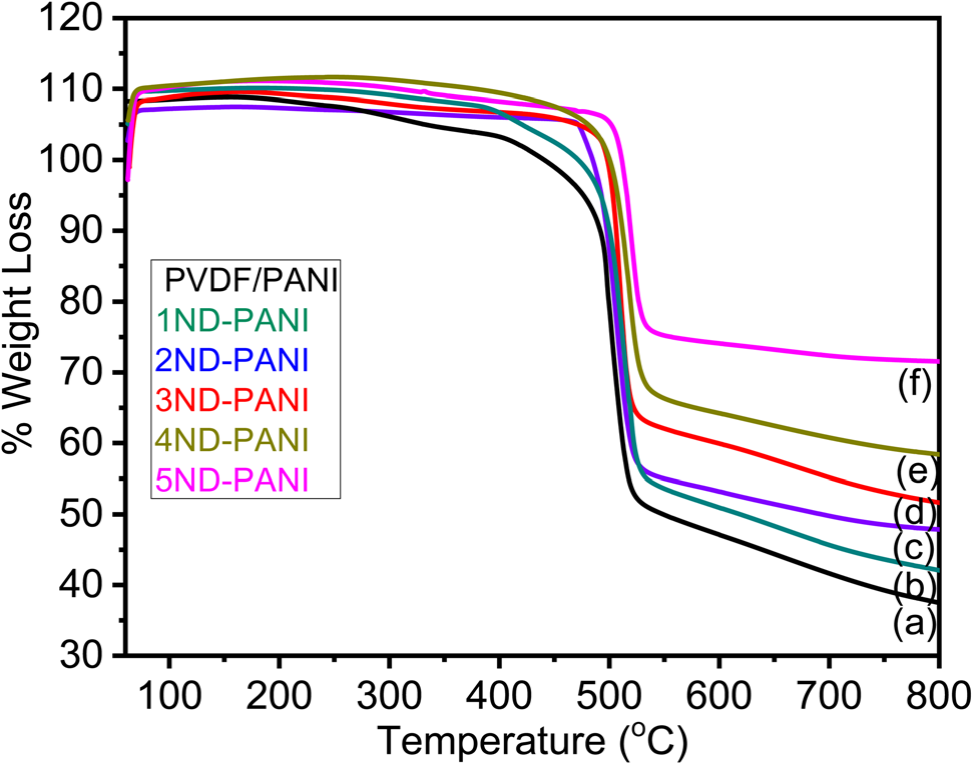
TGA graphs of (a) undoped PANI/PVDF polyblend (b) 1ND-PANI (c) 2ND-PANI (d) 3ND-PANI (e) 4ND-PANI (f) 5ND-PANI.

The TGA profile of undoped PVDF-PANI membrane and its composites with nanodiamonds is displayed in Fig. 4. These results clearly illustrate the increase in the thermal stability of the composite membranes with increasing nanodiamond content. The nanodiamonds owing to its significant thermal properties contribute thermal stability to the composite membranes. The TGA profile represents thermal decomposition as a one step process which is due to the evaporation of adsorbed gases, moisture and organic impurities which might be incorporated in the matrix during the synthesis process.

The temperature profile of percent weight losses (5%, 10%, and maximum) of ND-doped PANI/PVDF composite membranes measured during TGA analysis are presented in Table 1.

**Table 1:** Temperature profile of percent weight loss of undoped PANI/PVDF and NDs-based PANI/PVDF composite membranes

Sr. no.	Composition	Temperature in °C			Residue%
		5% wt loss	10% wt loss	Max. wt loss	
1	Undoped PANI/PVDF	406	440	540	48
2	1ND-PANI	408	469	542	50
3	2 ND-PANI	440	488	550	58
4	3ND-PANI	495	500	552	58
5	4 ND-PANI	495	501	556	60
6	5ND-PANI	501	512	560	65

5% weight loss appears in the temperature range of 406–501 °C, 10% weight loss takes place in temperature range of 440–512 °C, and maximum weight loss occurs between 540-556 °C whereas the residual amount of the compound varies from 48–65% for undoped PANI/PVDF and its ND-based composite membranes. The shift in weight loss and increase in residue value can be explained on the basis of an increase in interfacial interactions between the filler and the matrix materials with the increase in concentration of nanodiamonds.

### 3.5 Properties of ND-based PANI/PVDF composite membranes

#### 3.5.1 Porosity and shrinkage ratio

**Fig. 5:**
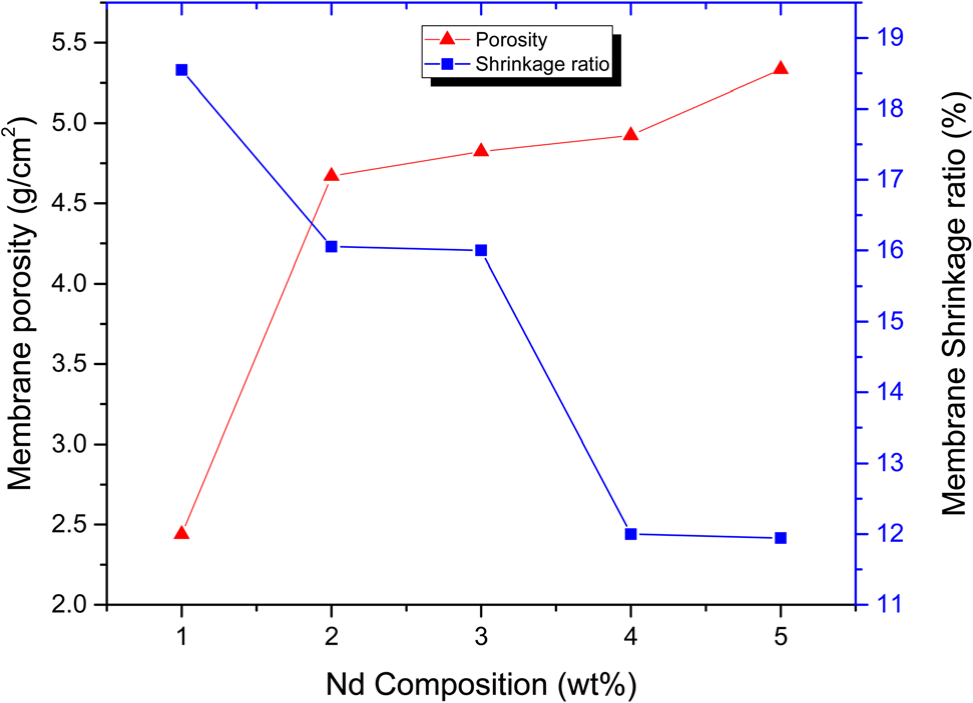
Porosity and shrinkage ratio of ND-based PANI/PVDF composite membranes.

Fig. 5 illustrates the membrane porosity measurements. It is evident from Fig. 5 that there is continuous increase in the porosity of the membranes as the concentration of NDs is increased from 1% to 5%. The increase in porosity from 2.41g cm^−2^ to 5.43g cm^−2^ by the addition of NDs in polyblend is attributed to the increasing hydrophilicity of the membranes as NDs are functionalized with hydroxyl (–OH) and carboxyl (–COOH) groups.^38,41^ Hence, the increase in hydrophilicity upon incorporation of NDs in the presence of PANI significantly contributes to the increase in composite membrane porosity. In our previous studies on ND-based PVDF membranes,

The membrane shrinkage ratio measurements are illustrated in Fig. 5 which shows that the % shrinkage ratio is decreased from 19% to 12% as the concentration of filler is increased from 1% to 5%. This is because the porosity of membranes is inversely related to its shrinkage ratio. The shrinkage ratio of ND-based PANI/PVDF composite membranes is found to be less than 20% in current study, one of the major requirement of wastewater treatment, implying effectives of investigated membranes in wastewater applications.^53^

#### 3.5.2 Solvent contents

In order to determine the membrane selectivity four different solvents were selected as water, methanol, ethanol, and propanol having dielectric constant (*ε*) 80.4, 33.1, 24.3, and 20.1, respectively. The results of solvent uptake/content of ND-based PANI/PVDF composite membrane is presented in Table 2. It is evident from Table 2 that the water content of the membranes has increased from 0.361 to 0.55 with increasing concentration of NDs from 1% to 5%, respectively. This is attributed to the enhancement of membrane porosity and hydrophilicity induced by addition of PANI and nanofillers which proves that incorporation of NDs in polyblend has significantly enhanced the permeability of composite membranes. Moreover, solvent content of these membranes has decreased from more polar solvent (water) to less polar solvents (propanol) depending upon their polarity. From results of solvent content, it is evident that solvent content of 5% ND composite membrane has maximum value for water and least for propanol, on other hand it has maximum value compared to 1% ND substitution level. The results indicate that incorporation of ND in PANI/PVDF polyblend has enhanced the hydrophobicity and porosity of the composite membranes.

**Table 2:** Solvent Contents of NDs-PANI/PVDF Composite membranes

Sample	Water	Methanol	Ethanol	Propanol
	*ε* (80.4)	*ε* (33.1)	*ε* (24.3)	*ε* (20.1)
1 ND-PANI	0.361	0.101	0.090	0.086
2 ND-PANI	0.488	0.150	0.135	0.113
3 ND-PANI	0.501	0.190	0.176	00.170
4 ND-PANI	0.541	0.220	0.199	0.189
5 ND-PANI	0.555	0.235	0.132	0.112

### 3.6 Antifouling properties of ND-based PANI/PVDF composite membranes

Pure water flux, rate of membrane filtration per square foot of its surface area, is the foremost criteria determining the fate of membrane for utilizing in the waste water treatment. As higher flux rate implies less membrane surface area requirement which in turn lowers the installation cost of treatment unit.^54^ In other words, the membrane permeability, flux rate per 1psi trans-membrane pressure, is the desirable feature of good quality membranes. Installation of the membrane in the treatment plant-assembly requires higher value of water flux at possibly low trans-membrane pressure that can satisfy the requirement. The water flux of ND-based PANI/PVDF composite membranes (mL cm^−2^ min^−1^) measured at 0.2 bar pressure is given in Table 3. As the concentration of NDs is increased from 1% to 5% the pure water flux increases from 8.0 mL cm^−2^ min^−1^ to 15.5 mL cm^−2^ min^−1^ due to enhancement of composite membrane porosity and hydrophilicity caused by NDs incorporation in the presence of PANI.

**Table 3:** Pure flux and fouling recovery ratio of ND-based PANI/PVDF composite blends microfiltration membranes

Sample	Pure water flux (mL cm^−2^ min^−1^)	Fouling recovery ratio (copper ions)	Fouling recovery ratio (zinc ions)
1ND-PANI	8.0	75.0	75.2
2ND-PANI	13.0	76.3	75.6
3ND-PANI	13.5	80.2	80.5
4ND-PANI	14.0	85.5	85.0
5ND-PANI	15.2	88.0	90.0

**Fig. 6:**
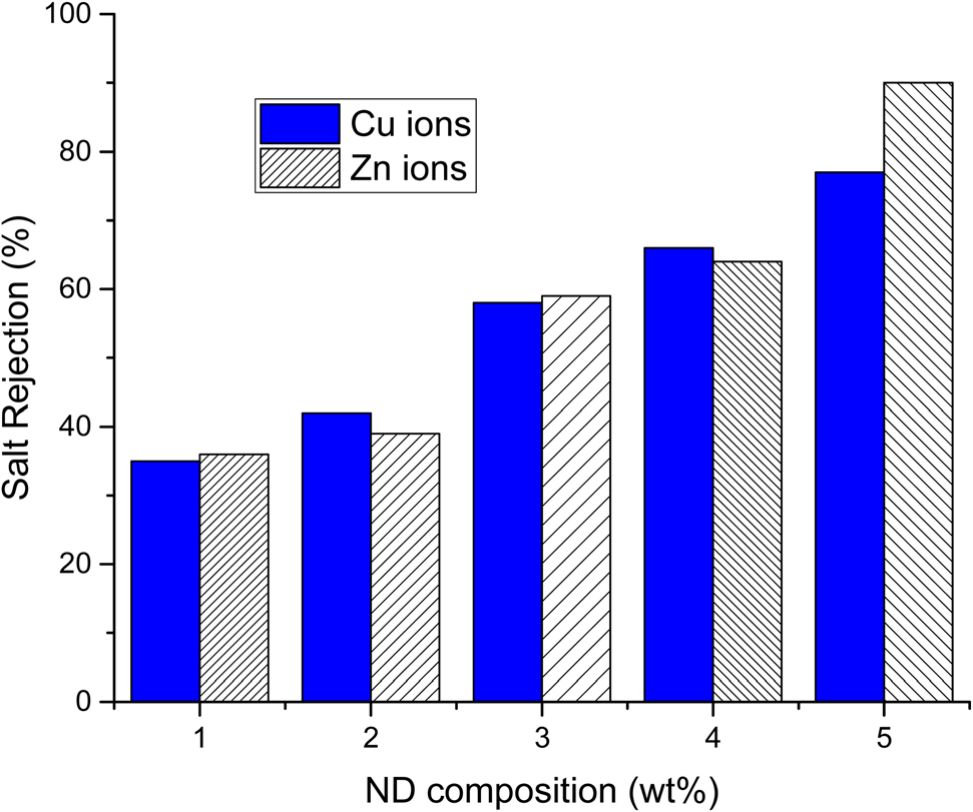
Salt rejection of ND-based PANI/PVDF composite blends microfiltration membranes.

The salt rejection (SR) and fouling recovery ratio (FRR) of ND-based PANI/PVDF composite membranes were tested against two heavy metals including copper acetate and zinc acetate solutions (0.1 M each), as illustrated in Fig. 6. As the concentration of NDs in polyblend is increased from 1% to 5% the % salt rejection increases from 35% to 77% and 36% to 90% for copper acetate and zinc acetate, respectively, which indicates that addition of ND has effectively enhanced the adsorptive capacity of membrane resulting in higher salt rejection.

Besides, FRR increases from 75% to 88% and 75% to 90%, as shown in Table 3, with increasing ND concentration from 1–5wt% for copper acetate and zinc acetate salts, respectively. These results established the fact that both the salt rejection and fouling recovery ratio of composite membranes are boosted up by increasing the content of ND filler. The membrane with higher loading of filer (5 wt% of NDs) showed the best results compared to 1% ND-PANI membrane.

## 4. Conclusion

A series of nanocomposite microfiltration membranes were fabricated by incorporating varying amounts of nanodiamond fillers (1–5% wt%) into blended polyaniline–polyvinylidene fluoride (PANI/PVDF) backbone *via* solution casting approach. Thermal analysis exhibits one-step decomposition of composite membranes with a maximum weight loss in the temperature range of 540–556 °C. XRD analysis exhibited the characteristic diffraction peaks at 20.5°, 36.71°, and 43.61° characteristics for ND-doped PANI/PVDF nanocomposite membrane exhibiting phase α → β transition of PVDF caused by ND inclusion. Membrane with higher loadings of filler (NDs) exhibited maximum porosity, water content, pure water flux, salt rejection and fouling recovery ratio. Thus, doping the PANI/PVDF membranes with NDs increases the efficiency of water filtration membranes. Furthermore, it is established that these membranes belong to the category of microfiltration as the porosity of the membranes corresponds to microfiltration membranes processes. In addition, the % shrinkage ratio was found less than 20% in all the cases which agrees with the recommended ratio for effective filtration to be less than 20%. These results indicate that PANI/PVDF composite microfiltration membranes impregnated with ND fillers can effectively be used in wastewater treatment.

## Author contributions

Asima Siddiqa: conceptualization, supervision, methodology, review & editing; Abdul Majid: data curation, formal analysis, writing original draft; Farhat Saira: review & editing, validation, investigation; Saima Farooq: review & editing, validation, visualization; Rumana Qureshi: project administration, resources; Sara Qaisar: project administration, resources.

## Conflicts of interest

The authors have no conflict of interest to be claimed.

## Supplementary Material
